# Selective mRNA Delivery to Activated Macrophages via Hyaluronic Acid-Functionalized Lipid Nanoparticles with Optimized PEGylation

**DOI:** 10.1021/acs.biomac.5c02390

**Published:** 2026-02-10

**Authors:** Mengyuan Cao, François Fay, Adrouchan Hotier, Séverine Domenichini, Lucile Alexandre, Christopher Ribes, Florence Gazeau, Hervé Hillaireau, Elias Fattal

**Affiliations:** † Institut Galien Paris-Saclay, UMR CNRS 8612, 27048 Université Paris-Saclay, 91400 Orsay, France; ‡ Institut Universitaire de France (IUF), 75005 Paris, France; § Plateforme MIPSIT-Ingénierie et Plateformes au Service de l’Innovation Thérapeutique, UMS-IPSIT Université Paris-Saclay, US 31 INSERM, UAR 3679 CNRS, 91400 Orsay, France; ∥ Laboratoire NABI NAnomédecine, Biologie extracellulaire, Intégratome et Innovations, 555089 Université Paris Cité, CNRS UMR8175, INSERM U1334 75006 Paris, France

## Abstract

Activated proinflammatory macrophages are associated with various inflammatory diseases, and due to their overexpression of the CD44 receptor, they may be targeted for therapy by hyaluronic acid (HA), its natural ligand. This study aimed to develop lipid nanoparticles (LNPs) functionalized with HA and stabilized with an optimized amount of poly­(ethylene glycol) (PEG) for targeted mRNA delivery to activated macrophages. Using microfluidic mixing, LNPs were produced with either 1.5% PEG (LNP_1.5%PEG_) or 0.5% PEG (LNP_0.5%PEG_). HA-coated LNPs (HA-LNPs) were prepared by postinsertion of an HA–DPPE conjugate, and changes in size and zeta potential demonstrated a successful and efficient HA coating, which was quantified by spectrofluorimetry and nanoscale flow cytometry. In vitro studies showed that HA-LNP_0.5%PEG_ exhibited better uptake in activated macrophages while maintaining mRNA transfection efficiency, whereas HA-LNP_1.5%PEG_ did not improve its uptake, suggesting that excessive PEG can hinder targeting. Overall, HA-LNP_0.5%PEG_ effectively delivered mRNA to activated macrophages with enhanced selectivity.

## Graphical abstract

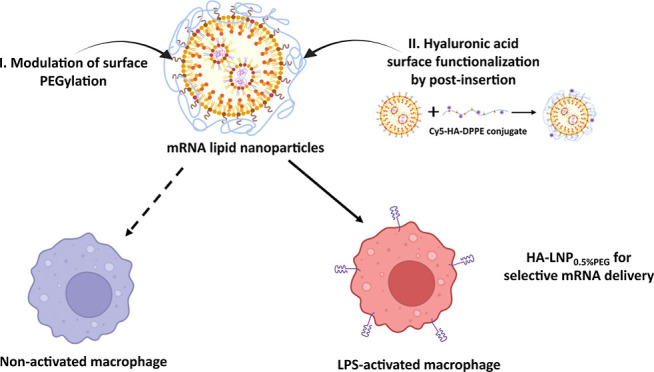

## Introduction

Macrophages are innate immune cells associated with multiple inflammatory diseases.^1,2^ In these diseases, following their interactions with pathogen-associated molecular patterns (PAMPs) and/or damage-associated molecular patterns (DAMPs), they produce proinflammatory cytokines (e.g., tumor necrosis factor α (TNF-α) and interleukin-6 (IL-6)) and chemokines (C–C motif chemokine ligand 2 (CCL-2) and (C-X-C motif) ligand 8 (CXCL-8)) that further drive their activation and polarization.^3,4^ Under such stimulation, tissue-infiltrated macrophages polarize to a proinflammatory state and thus secrete more proinflammatory molecules, exacerbating the inflammation. Therefore, targeting activated inflammatory macrophages has been considered as a potential strategy to address inflammatory disorders.^5^

Traditional approaches to treat inflammatory disorders, including therapeutic antibodies, broad-spectrum medications, transplantation, and stem cell therapies, face several hurdles, such as nonspecificity, physiological barriers, and immune rejection.^6,7^ Many studies support the idea that mRNA therapy should be tailored to encode and express essential proteins to restore or alleviate inflammation by delivering them to diseased cells (e.g., macrophages and neutrophils). Over the past decade, RNA therapeutics, particularly mRNA delivered by LNPs, have achieved remarkable breakthroughs and demonstrated significant clinical potential.^8^ However, owing to the complexity and heterogeneity of inflammatory environments, it is crucial to selectively target and deliver mRNA-LNPs to inflamed macrophages while minimizing their impact on healthy cells and tissues. For this purpose, various active targeting strategies have been developed to enhance cellular uptake, improve mRNA transfection efficiency, and facilitate cargo release in late endosomes without degradation in lysosomes.^9,10^

CD44 receptors are endocytic receptors widely expressed across various cell types, which are notably upregulated in activated proinflammatory macrophages,^11–13^ making them promising targets for LNP-mRNA delivery. HA is a natural glycosaminoglycan that serves as a ligand for CD44 with a notable affinity (dissociation constant (*K*_D_) in the micromolar range).^14^ HA has been widely explored for developing nanomedicines for active targeting, pain soothing, and joint lubrication in the regenerative and anti-inflammation domains.^14–17^

Various strategies have been developed to functionalize LNPs with HA, including electrostatic complexation (layer-by-layer assembly),^18^ direct chemical conjugation,^19,20^ and anchoring HA–lipid conjugate onto the LNP surface through pre- or postinsertion methods.^21–27^ While electrostatic complexation involves multiple steps and is unstable, direct chemical conjugation may induce cell damage due to the presence of residual reagents. Conversely, HA–lipid conjugates provide an easier way for HA coating thanks to the multiple reactive groups on HA (e.g., carboxyl, hydroxyl, and *N*-acetyl groups). However, the formulations of targeted LNPs need to be tailored and adapted to the specific scenario. For instance, PEGylated lipids are a double-edged sword in LNP formulations. They play a crucial role in maintaining the colloidal stability of LNPs^28–30^ and enhance the blood circulation time of LNPs administered by intravenous injection. On the other side, PEG also triggers specific immune responses, hinders the mRNA endosomal escape, and impairs the efficiency of targeting ligands.^16,31–35^ Therefore, when targeted mRNA-LNPs are designed for local delivery to inflammatory sites, it is essential to optimize the PEG content in the formulations to balance the colloidal stability and targeting efficiency of the LNPs.

In this study, we aim to functionalize mRNA-LNPs with HA–DPPE using pre- and postinsertion methods. Multiple features of HA-LNPs were characterized, including their size, zeta potential, mRNA encapsulation efficiency, morphology, and HA coating efficiency. Their cellular uptake, selectivity, and mRNA transfection efficiency were investigated in nonactivated and lipopolysaccharide (LPS)-activated RAW 264.7 macrophages.

## Materials and Methods

### Materials

HA (300–500 kDa) was purchased from Contipro (France). 1-Ethyl-3-(3-(dimethylamino)­propyl) carbodiimide hydrochloride (EDC), *N*-hydroxysuccinimide (NHS), triethylamine, 1,2-di­(9*Z*-octadecenoyl)-*sn*-glycero-3-phosphocholine (DOPC), and cholesterol were provided by Sigma-Aldrich. 1,2-Dihexadecanoyl-*sn*-glycero-3-phosphoethanolamine (DPPE) was purchased from Lipoid. Tert-butanol was purchased from CARLO ERBA Reagents. D-Lin-MC3-DMA was obtained from MedChemExpress. 1,2-Dimyristoyl-rac-glycero-3-methoxypolyethylene glycol-2000 (DMG-PEG_2000_) and 1,2-dioleoyl-*sn*-glycero-3-phosphoethanolamine-*N*-(lissamine rhodamine B sulfonyl) (ammonium salt) (18:1 Liss Rhod-PE) were provided by Avanti Polar Lipids, INC. Cyanine 5 (Cy5)-amine was purchased from Lumiprobe and CleanCap Firefly Luciferase (Fluc) mRNA (5moU) from TriLink.

### Synthesis and Characterization of HA–DPPE Conjugate

The HA–DPPE conjugate was synthesized, as described by Pandolfi et al.,^21^ with some modifications. Briefly, HA (molecular weight, *M*_W(Disaccharide unit)_: 379.3143 g/mol, 100 mg, 0.26 mmol) was dissolved in 20 mL of Milli-Q water. Then, EDC (50.5 mg, 0.26 mmol) and NHS (30.3 mg, 0.26 mmol) were added to the aqueous solution to activate the carboxylic groups of HA. DPPE (36.5 mg, 0.05 mmol) was dissolved in 20 mL of a 9:1 (v/v) mixture of *tert*-butanol and Milli-Q water, and 90 μL of triethylamine was added. The mixture was heated to 55 °C until completely dissolved. The DPPE solution was then added dropwise to the HA solution, and the resulting mixture was stirred at 60 °C for 6 h, followed by stirring at room temperature overnight. The mixture was dialyzed against Milli-Q water using a 10 kDa Spectra/Por dialysis bag (Fisher Scientific) for 48 h to remove excess reagents and then centrifuged twice at 1693 *g* for 30 min to completely remove the free DPPE by precipitation. Finally, the aqueous solution was lyophilized to obtain the solid HA–DPPE product. The overall yield of the reaction was estimated to be 35%.

The proton nuclear magnetic resonance (^1^H NMR) spectra of HA and HA–DPPE were recorded by using a Bruker Avance3 HD 400 spectrometer at 400 MHz. HA and HA–DPPE were dissolved in deuterium oxide (D_2_O). The DPPE substitution degree (SD_DPPE_) was calculated by using the following formula:

$${SD}_{DPPE}=\frac{{Area}_{1.3\;ppm\;of\;‐\;{CH}_{2}\;group\;on\;DPPE}}{{Area}_{2.0\;ppm\;of\;N‐acetyl\;proton\left(methyl\right)\;on\;HA}}$$

### Synthesis of the Cy5–HA–DPPE Conjugate

HA (300–500 kDa, 20 mg, 53 μmol, Contipro Inc.) was dissolved in 4 mL of Milli-Q water. Then, EDC (10.1 mg, 53 μmol) and NHS (6.1 mg, 53 μmol) were added to the aqueous solution to activate the carboxylic groups of HA. DPPE (7.3 mg, 3 μmol) and Cy5-amine (1.7 mg, 3 μmol) were dissolved in 4 mL of tert-butanol/Milli-Q water (9:1 v/v) in the presence of 18 μL of triethylamine and heated to 55 °C until completely dissolved. The DPPE and Cy5 solution was then added dropwise to the HA solution, and the resulting mixture was stirred at 60 °C for 6 h and then stirred at room temperature overnight. The mixture was dialyzed against Milli-Q water using a 10 kDa Spectra/Por dialysis bag for 48 h to remove excess reagents and then centrifuged twice at 1693 *g* for 30 min to completely remove the free DPPE by precipitation. Finally, the aqueous solution was lyophilized to obtain the solid Cy5–HA–DPPE product. The overall yield of the reaction is estimated to be 23%. The fluorescence of Cy5 was determined using a spectrofluorometer (HORIBA Scientific, Jobin Yvon Technology), and the concentration of Cy5 was calculated using a standard curve of Cy5. The degree of Cy5 conjugation was determined using the following equation:

$${Conjugation\;degree}_{Cy5}=\frac{m_{Cy5}}{m_{Cy5‐HA‐DPPE}}$$

### Nanoparticle Preparation by Microfluidic Mixing

LNPs were prepared by microfluidic mixing of an organic phase and an aqueous phase. In the organic phase, lipids were mixed in the following molar ratio with a fixed mass concentration of 2 mg/mL in absolute ethanol: D-Lin-MC3-DMA: DOPC: Cholesterol: DMG-PEG_2000_ = 50:10:38.5:1.5 mol % for LNP_1.5%PEG_ formulation, or 50:10:39.5:0.5 mol % LNP_0.5%PEG_ formulation. The aqueous phase consisted of 30 mM citrate buffer (pH 4) containing Fluc mRNA with an N/P ratio of 12. The organic and aqueous phases were loaded in 2.5 and 5 mL syringes (Terumo), respectively. The LNPs were assembled using two pumps (DARWIN Microfluidics, New Era Pump Systems, N°300) equipped with a microfluidic herringbone chip (DARWIN Microfluidics, ChipShop Herringbone mixer) and their respective cartridges, with a flow rate ratio of 3:1 (aqueous:ethanol) and a total flow rate of 4 mL/min. The herringbone mixer channel 3 was used, which has an inlet diameter of 0.3 mm and an outlet diameter of 0.6 mm, with a channel and chamber volume of 2.26 mm^3^. The LNPs were washed three times with phosphate-buffered saline (PBS) using Amicon Ultra-15 100 K tubes at 3600 *g* and 4 °C for 10 min to remove free ethanol and unassembled lipids.

HA-LNPs were prepared using either pre- or postinsertion methods. In the preinsertion method, 10% HA–DPPE (w/w of total lipid) was dissolved in the aqueous phase, which contained citrate buffer and mRNA. This was followed by nanoparticle formation through microfluidic mixing, as described above. In the postinsertion method, nanoparticles were first prepared and then incubated with 10% HA–DPPE (w/w of total lipid) at 37 °C for 2 h. After incubation, the nanoparticles were ultracentrifuged at 40,000 rpm, 4 °C for 2 h using an Optima LE-80K ultracentrifuge (Beckman COULTER, rotor 70.1.Ti, SN10 × 10E1129) to remove the free HA–DPPE fraction present in the supernatant (phase 2 shown in Figure S1) and eliminated using needle-equipped syringes. The nanoparticles were resuspended in PBS and stored at 4 °C until further use.

### Phospholipid Quantification

The DOPC concentration in P1 and P2 (Figure S1A) was quantified using Stewart’s method.^36^ Briefly, after ultracentrifugation, 100 μL of P1 or P2 was added to 600 μL of 0.1 M FeCl_3_ and 0.4 M NH_4_SCN, in addition to 600 μL of chloroform. After vortexing for 1 min, the lower chloroform phase was transferred to a quartz cuvette (Hellman, 10 mm), and its absorbance at 472 nm was measured using a UV/vis spectrometer (Lambda 25, PerkinElmer). The concentration of DOPC was calculated from a DOPC calibration curve (linear range 0–76.9 μg/mL; *R* ^2^ = 0.9953).

### Dynamic Light Scattering and Zeta Potential

The average hydrodynamic diameter, polydispersity index (PDI), and zeta potential of nanoparticles were assessed by dynamic light scattering using Zetasizer Ultra (Malvern) and a cuvette (DTS1070, Malvern) at 25 °C. The nanoparticles stored in PBS were diluted 100-fold in 1 mL of Milli-Q water or in a 1 mM NaCl solution for zeta potential measurement. Experiments were run in triplicate with an equilibration time of 60 s.

### Cryo-Transmission Electron Microscopy

For cryo-TEM analysis, the surface of a cryo-TEM carbon-coated copper grid (MultiA Cu300 mesh grid, Quantifoil) was hydrophilized using the ELM Glow Discharge System (Cordouan Technologies) under 2 mbar of pressure and 4 mA of current for 40 s in an air atmosphere to facilitate the spread of LNP samples evenly on the grid. 3 μL of 20 mg/mL of LNP or HA-LNP (0.5% or 1.5% PEG, n/n) sample droplet was deposited on the grid. Samples were prepared using a semiautomated vitrification system (Vitrobot Mark IV, ThermoFisher) at 20 °C and 100% humidity, with a blot time of 4.5/3.5 s and a blot force of 2. The samples were then plunged into liquid-nitrogen-cooled ethane. Cryo-TEM images were observed and acquired using a Glacios 2 electron microscope (Thermo Fisher), operating at 200 kV with a magnification of 73,000× and a pixel size of 0.1928 nm. Sample preparation and data acquisition were performed at the cryo-electron microscopy platform in the Institute for Integrative Biology of the Cell (I2BC).

### Agarose Gel Electrophoresis

To assess mRNA encapsulation, 3 μL of mRNA-encapsulated LNPs or HA-LNPs was mixed with 17 μL of PBS or 0.1% (v/v) TritonX-100 and 4 μL of loading buffer (6 ×; G2526, Sigma-Aldrich). Samples were then loaded in the wells of 1% agarose gel (Sigma-Aldrich) containing 0.5 μg/mL of ethidium bromide (Bio-Rad) in 1 × *Tris*-borate-ethylenediaminetetraacetic acid (TBE) buffer (Invitrogen, Thermo Fisher Scientific) at 120 V for 60 min. An mRNA standard set (0.2–0.0002 mg/mL, Fluc mRNA) was run simultaneously to generate a calibration curve, from which the mRNA concentrations were calculated. To quantify the mRNA encapsulation, two equations were used.

Encapsulation fraction (EF) was used to describe the percentage of RNA that was encapsulated by LNPs in the loaded samples, reflecting the mRNA encapsulation ability of LNPs:

$$EF=\frac{{{\left[mRNA\right]}_{Triton}-\left[mRNA\right]}_{Non‐Triton}}{{\left[mRNA\right]}_{Triton}}$$

Encapsulation yield (EY) represents the percentage of input RNA from the aqueous phase during microfluidic mixing that is successfully encapsulated in the final LNPs, reflecting the amount of mRNA lost during the preparation process, which was 20–40 % in this study:

$$EY=\frac{m_{Triton\;mRNA}\times EF}{m_{Input\;mRNA}}$$

### HA Incorporation Efficiency by Spectrofluorimetry

To prepare fluorophore-labeled HA-LNPs, LNPs were coincubated with an increasing amount of HA–DPPE (0, 3, 8, 10, 20, 30, 50%, w/w of total lipid) and Cy5–HA–DPPE (30% of HA–DPPE, i.e., 0, 0.9, 1.5, 2.4, 3.0, 6.0, 9.0, 15.0%, w/w of total lipid) at 37 °C for 2 h. After incubation, the nanoparticles were ultracentrifuged at 40,000 rpm and 4 °C for 2 h using an Optima LE-80K ultracentrifuge (Beckman Coulter, rotor 70.1.Ti, SN 10 × 10E1129). The transparent phase (P2 shown in Figure S1) was carefully removed using needle-equipped syringes, and the remaining nanoparticles in the opaque phase (P1 shown in Figure S1) were resuspended in PBS. The fluorescence of the two phases was determined using a spectrofluorimeter (HORIBA Scientific, Jobin Yvon Technology), and the concentration of Cy5 was calculated from a calibration curve. The HA coating efficiency was determined using the following equation:

$$HA\;incorporation\;efficiency=\frac{m_{Cy5\;in\;P1}}{m_{Cy5\;in\;P1}+m_{Cy5\;in\;P2}}$$

### Nanoscale Flow Cytometry

Nanoscale flow cytometry analysis was performed using the Flow NanoAnalyzer (N30E, NanoFCM Co., Ltd.) according to the manufacturer’s instructions. The instrument was calibrated with a mixture of four sizes of silica beads (S16M-Exo, 68, 91, 113, and 155 nm) for size measurements and with QC 250 nm silica beads for concentration measurements. LNPs were labeled by incorporating 1% (n/n) Rhod-PE during microfluidic mixing. To prepare fluorophore-labeled HA-LNPs, LNPs were coincubated with 3% (w/w) Cy5–HA–DPPE and 10% (w/w) HA–DPPE, followed by purification via ultracentrifugation and concentration in PBS to 10^12^ particles/mL. Before measurement, the samples were diluted 1000-fold in PBS. Two lasers, emitting at 488 and 640 nm, were illuminated simultaneously to excite the fluorophore. A bandpass filter at 580 ± 40 nm was used to detect the signal from Rhod-PE-labeled LNPs, corresponding to the 488 nm excitation. A bandpass filter at 670 ± 30 nm was used to detect the signal from Cy5–HA–DPPE-labeled HA-LNPs, corresponding to the 640 nm excitation. Data were processed and analyzed using FlowJo software.

### Cell Culture

Murine macrophage cell line RAW 264.7 (ATTC TIB-71), obtained from ATCC, was cultured in Dulbecco’s modified Eagle’s medium (Gibco) supplemented with 10% heat-inactivated fetal bovine serum (Gibco), 50 U/mL penicillin, and 50 U/mL streptomycin at 37 °C in a 5% CO_2_ environment and 95% humidity. Cells were split when they reached approximately 80% confluence at a 1:10 ratio using a scraper. The cell culture was performed for no more than 20 passages.

### CD44 Expression Level

RAW 264.7 macrophages were seeded onto 12-well culture plates at 3 × 10^5^ cells/mL of medium for 24 h. The cells were treated with 100 ng/mL of LPS (*Escherichia coli* O55:B5, L2880, Sigma-Aldrich) for 24 h to activate the macrophages.

The expression of CD44 was evaluated using flow cytometry with mouse antihuman CD44 antibodies (Cat#: 559942, BD Pharmingen). The nonactivated or activated macrophages were harvested using scrapers and resuspended in 50 μL of Stain Buffer (BD Pharmingen). Cells were pretreated with 2 μL of mouse Fc blocker (Cat#: 553142, BD Pharmingen) for 5 min and then incubated with CD44 antibodies or isotype antibodies (10 μL, 1:6) at 4 °C in the dark for 30 min. After incubation, the cells were washed three times with 1 mL of Stain Buffer, centrifuged at 300*g* and 4 °C for 5 min, and then resuspended in 300 μL of Stain Buffer. Cell fluorescence was acquired using a BD Accuri C6 Cytometer (BD Biosciences, USA), with excitation at 640 nm and detection using filter 4 (675/25 nm). Data were analyzed using FlowJo software, employing a gating strategy illustrated in Figure S3.

### Cell Uptake Assay

RAW 264.7 macrophages were seeded onto 12-well culture plates at 3 × 10^5^ cells/mL of medium for 24 h. The cells were treated with 100 ng/mL LPS to activate macrophages for 24 h. Then, the medium was replaced by 1 mL of fresh medium, and the cells were incubated with 18:1 Liss Rhod PE (1%, n/n)-labeled LNPs or HA-LNPs (2 μM Rhod PE, 1.5% and 0.5% PEG formulations) for 4 h. In competitive binding studies, macrophage cells were preincubated with 0.5 mg/mL of free HA (300–500 kDa) for 1 h. Then, the Rhod PE-labeled nanoparticles were added and incubated with the cells for 4 h. After incubation, the cells were washed twice with PBS, harvested using cell scrapers, and resuspended in 300 μL of Stain Buffer. The cell mean fluorescence intensity (MFI) was acquired using a BD Accuri C6 Cytometer (BD Biosciences, USA), with excitation at 488 nm and detection using filter 2 (585/40 nm). Data were analyzed by using FlowJo software. Dead cells and debris were excluded on the basis of forward-scatter (FSC) and side-scatter (SSC) measurements. Their mean fluorescence intensity (MFI) was normalized using blank cells (fold-change).

### Cell Internalization by Confocal Laser Scanning Microscopy

The cellular uptake and internalization of LNP_0.5%PEG_ and HA-LNP_0.5%PEG_ have been evaluated in both nonactivated and activated macrophages. RAW264.7 cells were seeded at a density of 3 × 10^5^ cells/mL in a 6-well plate in which 1.5 H glass coverslips were preplaced. After 24 h, cells were activated with 100 ng/mL of LPS for an additional 24 h. Next, the macrophages were treated with Rhod PE-labeled LNP or HA-LNP with a final rhodamine concentration of 2 μM for 4 h at 37 °C in the dark. After incubation, the cells were washed three times with PBS and fixed with 4% (w/v) paraformaldehyde (PFA) for 20 min. After rewashing the coverslips, 25 μL of antifade mounting medium containing 4,6-diamidino-2-phenylindole (DAPI, VECTASHIELD, Cat#H-1200, Vector Laboratories) was added to the cells. The coverslip was placed on the microscope slides and sealed with nail polish. The images were captured using an inverted TCS SP8 (gated-STED) confocal microscope (Leica Microsystems, Germany), an HC PL APO CS2 63×/1.04 oil immersion objective lens, and Leica LAS X (v3.5.5) software. The microscope was equipped with a 405 nm diode laser for DAPI staining (cyan channel) and a white light laser set at 560 nm for rhodamine (red channel), and fluorescence emissions were collected using a sequential mode with a variable beam-splitter set, respectively, between 415 and 470 and 570–631 nm with a photomultiplier tube (PMT) for DAPI, an internal hybrid detector (HyD) for rhodamine, and PMT-trans for transmitted light. The detection offset was chosen to a small number of zero-value pixels, and detector gains were set to optimize the dynamic range (12 bits images) while ensuring minimal saturated pixels from a condition of interest assumed to be the strongest in signal and were kept for all acquisitions of the same analysis. The pinhole was set at 1.0 Airy unit (optical thickness of 0.89 μm). For each coverslip, a minimum of 5 different fields were acquired. Images were processed (e.g., merging) using LAS X software (Leica Microsystems, Germany) only to enhance the image display by adjusting brightness and contrast settings identically for each image presented. Raw data were available.

### In Vitro Transfection Efficiency

RAW 264.7 macrophages were seeded onto 96-well plates at 3 × 10^5^ cells/mL medium for 24 h. To activate macrophages, the cells were treated with 100 ng/mL of LPS for 24 h. Cells were transfected with Fluc mRNA encapsulated in LNPs or HA-LNPs at varying mRNA quantities (25, 50, 100 ng) in 200 μL of medium for 24 h. The luminescence radiance was determined using a Pierce Firefly Luciferase Glow Assay Kit (Thermo Scientific) by an EnVision Multilabel Reader 2103 (PerkinElmer, Revvity) and normalized by the protein content determined by the BCA kit (Pierce, Thermo Scientific).

### In Vitro Cytotoxicity Assay

Cell viability was assessed using a thiazolyl blue tetrazolium bromide (MTT) assay. RAW 264.7 macrophages were seeded onto 96-well plates at 3 × 10^5^ cells/mL of medium for 24 h. To activate macrophages, the cells were treated with 100 ng/mL of LPS for 24 h. The phospholipid concentration was determined based on Stewart’s method.^36^ Then, LNP formulations were diluted in the culture medium to obtain a total lipidic concentration ranging from 0.5 to 50 μg/mL. Cells were allowed to grow for an additional 24 h. After incubation, 20 μL of MTT solution (5 mg/mL) was added to 200 μL of medium, and the cells were incubated at 37 °C for 30 min. Then, the medium was replaced with 100 μL of dimethyl sulfoxide (DMSO), and the sample was shaken in the dark for 5 min. The absorbance of each well was measured at 570 nm using a Labtech LT-5000 Plate Reader. Cell viability was normalized to that of the blank cells (cells treated with PBS).

### Statistical Analysis

The data were analyzed for statistical significance using a two-way ANOVA. All data are expressed as mean ± SD. A P-value less than 0.05 was considered statistically significant. Data were analyzed using GraphPad PRISM software.

## Results and Discussion

### Synthesis and Characterization of HA–DPPE

To facilitate the functionalization of the surface of LNPs with HA, we conjugated HA to DPPE lipids, allowing for their insertion into the lipid layer of LNPs. The HA–DPPE conjugate was synthesized using EDC/NHS chemistry by coupling the amine group of DPPE to the carboxylic group of HA, forming an amide bond (Figure 1A).

**Figure 1:**
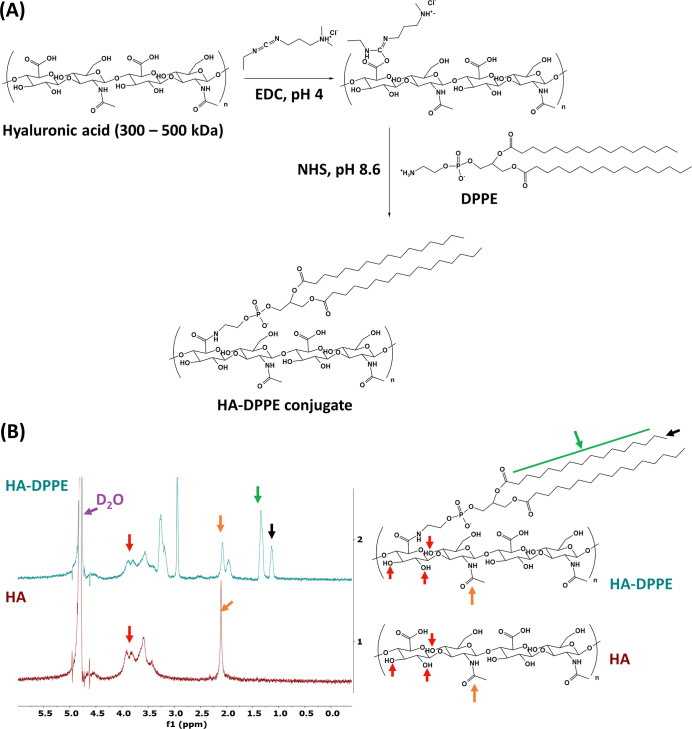
Synthesis and characterization of the HA–DPPE conjugate. (A) Schematic representation of the synthesis of HA–DPPE. (B) ^1^H NMR spectra of HA–DPPE conjugates and HA and the corresponding chemical structures in deuterium oxide (D_2_O), highlighting the methylene group of DPPE (green arrow), the methyl group of DPPE (black arrow), the *N*-acetyl proton (methyl) of HA (orange arrow), and the hydroxyl groups of HA (red arrow).

The conjugation between HA and DPPE was validated by ^1^H NMR (Figure 1B). The ^1^H NMR spectrum of HA–DPPE contains not only the peaks of hydroxyl groups (red arrows, 3.5 to 4 ppm) and *N*-acetyl proton (methyl, orange arrows, 2.0 ppm) of HA but also the peaks corresponding to the methylene group of the DPPE aliphatic chain (green arrow) at 1.3 ppm and the terminal methyl group (black arrow) at 1.1 ppm, indicating the successful conjugation of some DPPE to HA. Using the method reported by Pandolfi et al.,^21^ the substitution degree of DPPE molecules grafted to HA was calculated using the ratio between the area of the peak of the methylene group on DPPE at 1.3 ppm and the area of the peak of the *N*-acetyl proton group on HA at 2.0 ppm, which was between 9% and 13%.

### Formulation of LNPs with Variable PEG Coating and HA Incorporation

The LNP formulations were produced using microfluidics (Figure 2) with a mixture of D-Lin-MC3-DMA, DOPC, cholesterol, and DMG-PEG_2000_. We selected D-Lin-MC3-DMA because it is a Food and Drug Administration (FDA)-approved ionizable lipid for RNA therapeutic delivery exhibiting minimal immunogenicity and proinflammatory effects, which is important in designing anti-inflammatory nanomedicines.^37^ DOPC was chosen as a structural lipid as it exhibited better transfection efficiency in vitro with relatively less proinflammatory effects.^38^ Cholesterol was used for stabilizing the LNPs. Traditional mRNA-loaded LNPs commonly utilize PEG_2000_ (1.5%, n/n) to enhance the colloidal stability of LNPs and prolong their systemic circulation, particularly after intravenous injection. However, PEGylated lipids have been associated with potential immunogenicity. They also tend to impede cellular uptake, mRNA endosomal escape, and targeting efficiency due to steric hindrance.^16,31,32,34^ Therefore, we first investigated the decrease in PEG content at the surface of LNPs to reduce potential immunogenicity, enhance cellular uptake, and potentially minimize the impact on the interaction between HA and CD44. We initially attempted to remove DMG-PEG_2000_ from the formulation and replace it with HA–DPPE (up to 50% w/w) or select a shorter PEG (1 kDa). However, all attempts resulted in LNP aggregation (data not shown). It was still possible to reduce the DMG-PEG_2000_ proportion from 1.5% (the conventional ratio) to 0.5% (n/n) while maintaining LNPs’ colloidal stability (Table 1), therefore offering the possibility to modulate the PEGylation of LNPs from 0.5% to 1.5% (the corresponding formulations are named LNP_0.5%PEG_ and LNP_1.5%PEG_ in the following). The selected N/P ratio was 12, instead of the current value of 6. This ratio was chosen to provide high transfection efficiency, better stability of entrapped mRNA, and, most importantly, a reduced particle size.^39^ Indeed, reducing the molar ratio of PEGylated lipids increased the particle diameter (Table 1). A further reduction of the N/P ratio would have significantly altered the LNP structure, potentially leading to an additional increase in size and affecting cellular uptake.

**Figure 2:**
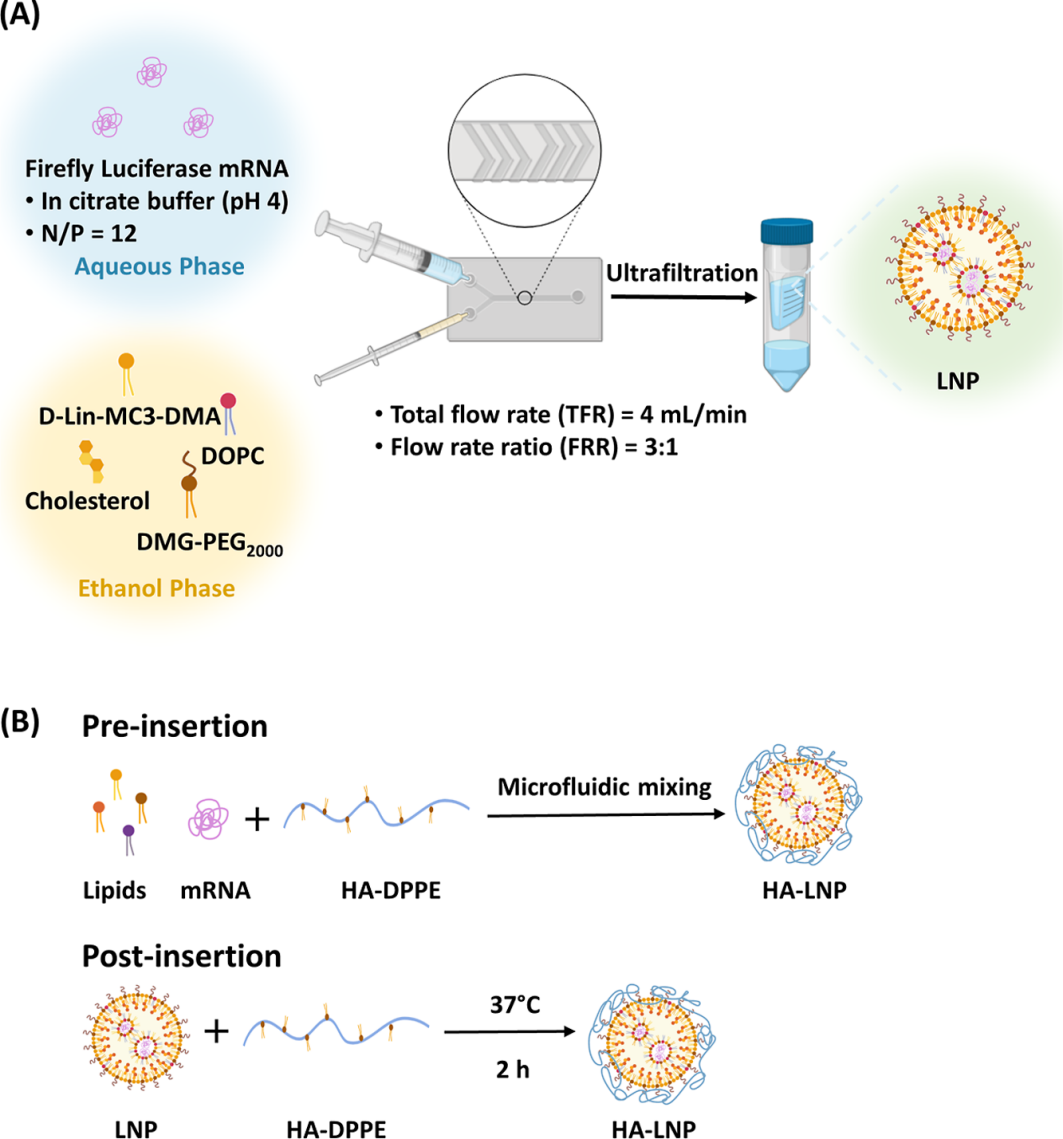
Schematic representation of the preparation of HA-coated lipid nanoparticles. (A) Preparation of lipid nanoparticles by microfluidic mixing through a herringbone microfluidic chip. (B) Pre- and postinsertion methods to coat HA–DPPE on LNPs.

**Table 1:** Colloidal Characterization of LNPs^a^

PEG %	name	HA coating method	*Z*-average (nm)	PDI	zeta potential (mV)	mRNA encapsulation fraction (%)
1.5%	LNP_1.5%PEG_	no HA	155 ± 21	0.14 ± 0.03	–5 ± 2	95 ± 7
	HA-LNP_1.5%PEG_	preinsertion	190 ± 8	0.24 ± 0.02	–14 ± 4	85 ± 7
	HA-LNP_1.5%PEG_	postinsertion	177 ± 12	0.235 ± 0.007	–34 ± 1	81 ± 9
0.5%	LNP_0.5%PEG_	no HA	256 ± 25	0.21 ± 0.06	3 ± 4	98 ± 2
	HA-LNP_0.5%PEG_	postinsertion	293 ± 31	0.24 ± 0.03	–32 ± 2	98.3 ± 0.6

a Particle size (mean hydrodynamic diameter, *Z*-average) and polydispersity index (PDI) were determined by dynamic light scattering, and zeta potential (in 1 mM NaCl) was measured by electrophoretic light scattering. mRNA encapsulation in LNPs was assessed by gel electrophoresis (*n* = 3).

The functionalization of LNPs with HA was then investigated by pre-or postinsertion of HA–DPPE (Figure 2). In both cases, purification methods to remove free HA–DPPE are challenging. Ultrafiltration is limited by the high MW of HA (300–500 kDa), and size exclusion chromatography is inadequate due to its low yield and poor purification efficiency.^40^ Ultracentrifugation was, therefore, applied to purify the free HA from HA-coated nanoparticles. Interestingly, for all formulations, ultracentrifugation did not result in separating a pellet from a supernatant but rather in a phase separation into an upper opalescent phase (P1 in Figure S1A) above a clear one (P2). We hypothesized that most nanoparticles were present in P1. To confirm this, we analyzed samples from both P1 and P2 using agarose electrophoresis and phospholipid quantification. In the agarose image (Figure S1B), a clear mRNA band appeared in the P1 sample after Triton X-100 treatment, indicating that intact nanoparticles containing mRNA were present in the opaque phase P1, which could be subsequently broken by Triton, releasing entrapped mRNA. In addition, the band of P1 after Triton X-100 treatment was far more intense than that of P2, suggesting that most LNPs were concentrated in P1. The phospholipid quantification further supported this assumption (Figure S1C). Using the phospholipid assay described by Stewart,^36^ we determined the phospholipid distribution between the two phases and found that 93% of DOPC was concentrated in P1. These results confirm that most of the LNPs were floating and concentrated in the upper phase after ultracentrifugation. Cryo-TEM analysis showed that LNPs and HA-LNPs produced by microfluidic mixing exhibited an internal structure predominantly composed of lipids rather than an aqueous solution, which may account for their lower overall density (Figure S2). Since the densities of nucleic acids are higher than that of lipids, the measured density is directly proportional to the loading of nanoparticles. Hence, different loading levels will produce particles with a variable density and partial specific volume. Henrickson et al. measured the nucleotide drug loading in LNPs with density-matching analytical ultracentrifugation.^41^ They found that under certain buffer conditions, empty LNPs float, while LNPs with an N/P ratio of 6 also float. In contrast, LNPs formulated at an N/P ratio of 1, which have higher RNA loading, sediment.

In the case of traditional 1.5% PEG formulations, LNPs had a mean hydrodynamic diameter of around 150 nm with a low polydispersity index, and their zeta potential was close to neutral (Table 1). After coating nanoparticles with HA using the postinsertion method, their zeta potential decreased significantly to −30 mV, indicating the successful insertion of HA. In contrast, HA-LNPs prepared by preinsertion exhibited a neutral or slightly negative zeta potential. One explanation is that HA, solubilized in citrate buffer (pH 4) during microfluidic mixing, is negatively charged due to deprotonation of its carboxylic groups (p*K*_a_ ∼ 3). At this pH, the positively charged D-Lin-MC3-DMA may interact electrostatically with HA, leading to its coencapsulation with mRNA inside the LNPs, and less HA appears on the surface of the nanoparticles. Therefore, we selected the HA postinsertion method for HA functionalization.

In the case of formulations containing 0.5% PEG, LNPs exhibited a mean diameter of around 250 nm (with mRNA) with a neutral zeta potential (confirming the importance of PEG in stabilizing and compacting the nanoparticles). Once coated with HA by the postinsertion method, their size increased to approximately 300 nm (Table 1), and their zeta potential decreased to around −30 mV, indicating successful HA coating.

In addition, Cryo-TEM images revealed that HA-LNPs prepared by postinsertion methods exhibited more irregular boundaries or diffuse shells compared to uncoated LNPs, suggesting again the presence of HA coating (Figure S2). Moreover, electron-dense cores embedded in the membrane near the edge of nanoparticles were supposed to be the encapsulated mRNA. Such morphology resembles the “emulsion-like LNP” model, which has been reported to preferentially remain at injection sites, thereby reducing the off-site immune responses.^42^ Additionally, such structures may also facilitate the release of mRNA upon cellular interaction. All nanoparticles had a high mRNA encapsulation fraction of >90%, confirming their high cargo-loading ability.

### Fluorescent Labeling and Quantification of HA Coating Efficiency on LNPs

We first employed a fluorescence-based method to further characterize the efficiency of HA coating on the nanoparticles. Cy5–HA–DPPE was synthesized (Cy5 conjugation degree = 10%) for the functionalization and labeling of the LNPs (Figure 3A). Both HA-LNP_1.5%PEG_ and HA-LNP_0.5%PEG_ formulations exhibited similar HA incorporation efficiencies of approximately 20% (Figure 3B). The HA incorporation efficiency reached a plateau for HA–DPPE ratios above 10% (Figure 3C). Therefore, 10% HA–DPPE was chosen for the HA-LNP preparation. Nanoscale flow cytometry was further used to investigate the homogeneity of the HA coating within the LNP samples. HA-LNP_0.5%PEG_ samples were labeled with 1% (n/n) of rhodamine-PE (Rhod) and coated with 3% (w/w) of Cy5–HA–DPPE. A 580 ± 40 nm detection was used to gate a Rhod-positive population (79.4%) identified as LNPs and a 670 ± 30 nm detection for Cy5-positive events. 93 ± 1% of the gated Rhod-positive events (referred to as LNPs) were also Cy5-positive, showing that most LNPs were coated with HA, confirming the efficient coating property of HA–DPPE by postinsertion (Figure 3D).

**Figure 3:**
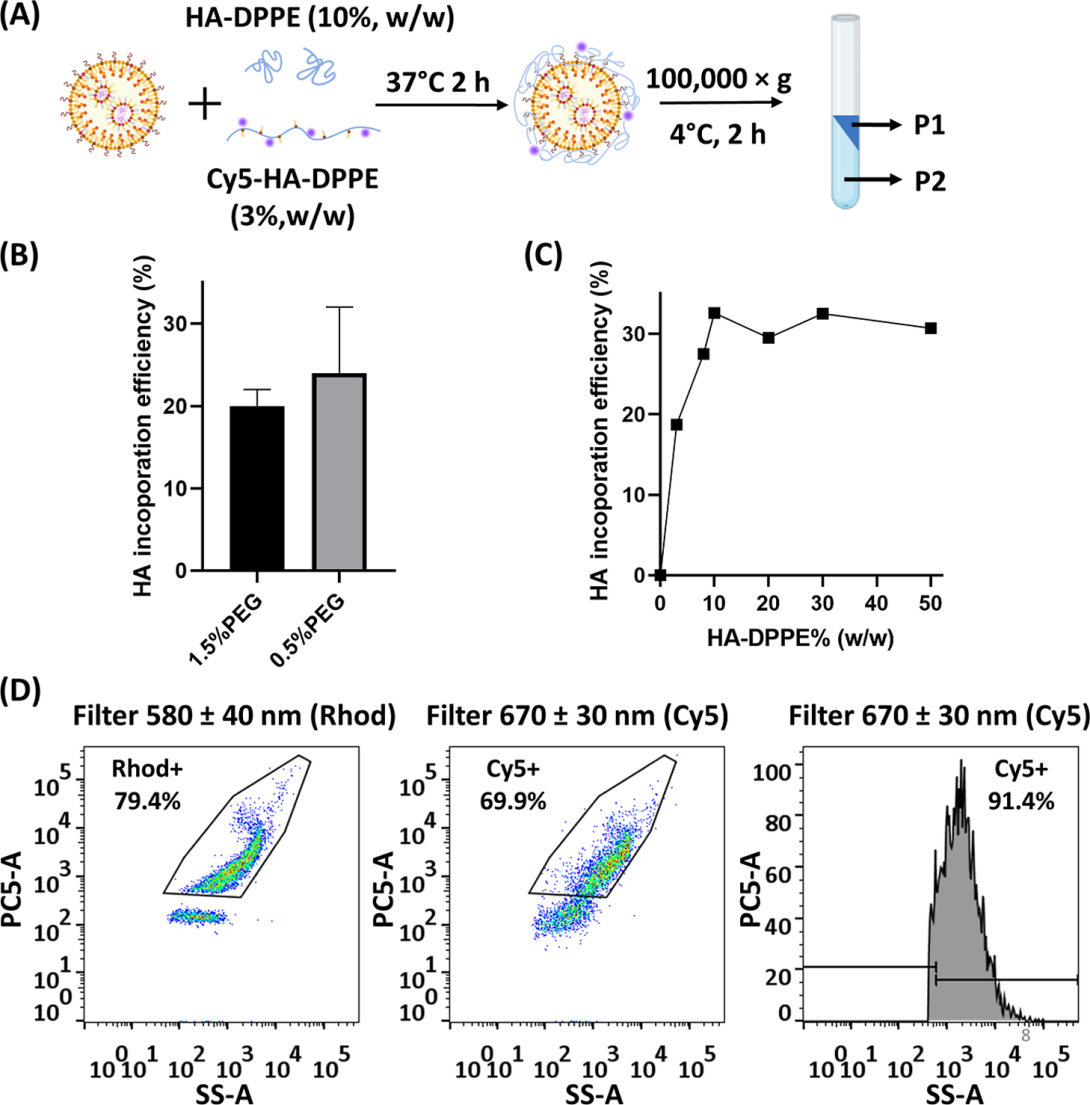
HA-coating efficiency determined by the fluorescence-based method and nanoscale flow cytometry. (A) Illustration of Cy5–HA–DPPE coating strategy on LNPs. (B) HA incorporation efficiency on LNP_1.5%PEG_ and LNP_0.5%PEG_ determined by the fluorescence-based method. (C) HA–DPPE incorporation efficiency on LNP_0.5%PEG_ using different HA–DPPE ratios. (D) HA–DPPE coating efficiency determined by nanoscale flow cytometry. Left: gating strategy for Rhod-positive population to gate LNPs using a bandpass filter at 580 ± 40 nm; middle: the same gating strategy applied using a bandpass filter at 670 ± 30 nm for the detection of Cy5–HA-labeled LNPs; right: histogram of gated LNP population under excitation at 670 ± 30 nm for Cy5-positive population detection.

### HA-LNP_0.5%PEG_ Selectively Targeted CD44-Overexpressing Macrophages

Cellular uptake studies of LNPs and HA-LNPs were performed on nonactivated or LPS-activated RAW 264.7 macrophage cells. The high expression of CD44 receptors in LPS-activated macrophages was confirmed by flow cytometry (Figure 4).

**Figure 4:**
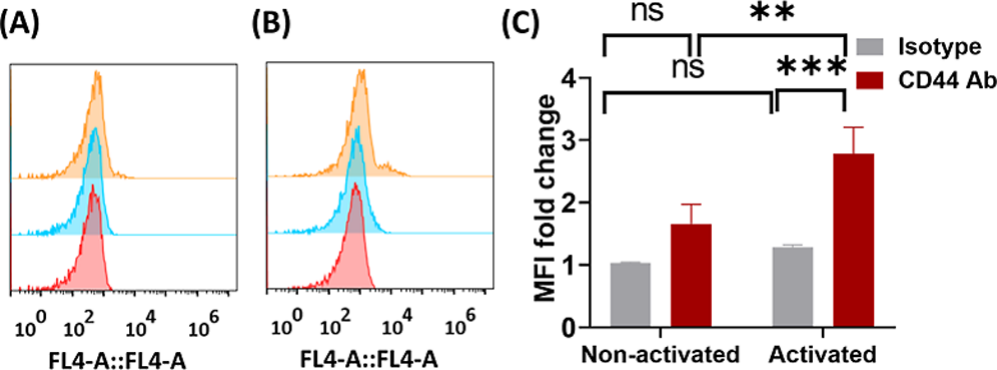
CD44 expression in macrophages by flow cytometry. (A) CD44 expression profile in nonactivated macrophages. (B) CD44 expression profile in activated macrophages. Red: cell controls; blue: isotype control antibody-treated cells; orange: anti-CD44 antibody-treated cells. (C) Quantitative representation of CD44 expression level in nonactivated macrophages and activated macrophages. ** = *p* < 0.01, *** = *p* < 0.001 using two-way ANOVA. *N* = 3.

Rhod-labeled LNPs or HA-LNPs (1.5% or 0.5% PEG) were incubated with nonactivated (CD44 basal expression) or activated macrophages (high CD44 expression), and their cellular uptake was investigated using flow cytometry. As shown in Figure 5, LNP_1.5%PEG_ and HA-LNP_1.5%PEG_ exhibited no significant difference in cellular uptake by either nonactivated or activated macrophages (Figure 5A,B). This pattern may be due to the interference between PEG and HA, as both molecules are long-chain polymers that can hinder or entangle with each other, thereby hiding the recognition sites of HA for CD44 receptors and impairing the CD44-targeting ability of HA-LNPs. This hypothesis is consistent with previous studies showing that PEG removal from liposomes significantly enhances cellular uptake in proinflammatory macrophages.^16^ When the PEG content was reduced to 0.5%, HA-LNPs showed decreased uptake by nonactivated macrophages but significantly enhanced uptake by activated macrophages, which was not the case for the uncoated LNPs. These results demonstrated the selective targeting ability and improved cellular uptake of the HA-LNP_0.5%PEG_ formulation. In addition, when the cells were preincubated with free HA to block the CD44 receptors, the internalization of HA-LNP_0.5%PEG_ in activated macrophages was significantly reduced (Figure 5D). In contrast, in nonactivated macrophages, HA preblocking had no impact on the cellular uptake of HA-LNP_0.5%PEG_, but it slightly increased the uptake of LNP_0.5%PEG_ (Figure 5C). This observation could be attributed to changes in the internalization pattern or the activation of alternative uptake pathways, leading to enhanced nonspecific uptake.^43,44^ Nevertheless, the HA competitive studies demonstrated that the cellular uptake of HA-LNP_0.5%PEG_ was primarily mediated by the HA–CD44 interaction and selectively targeted LPS-activated macrophages instead of normal macrophages. These results also highlighted the influence of fine-tuning the proportion of PEG on modulating the targeting ability of ligand-functionalized nanoparticles.

**Figure 5:**
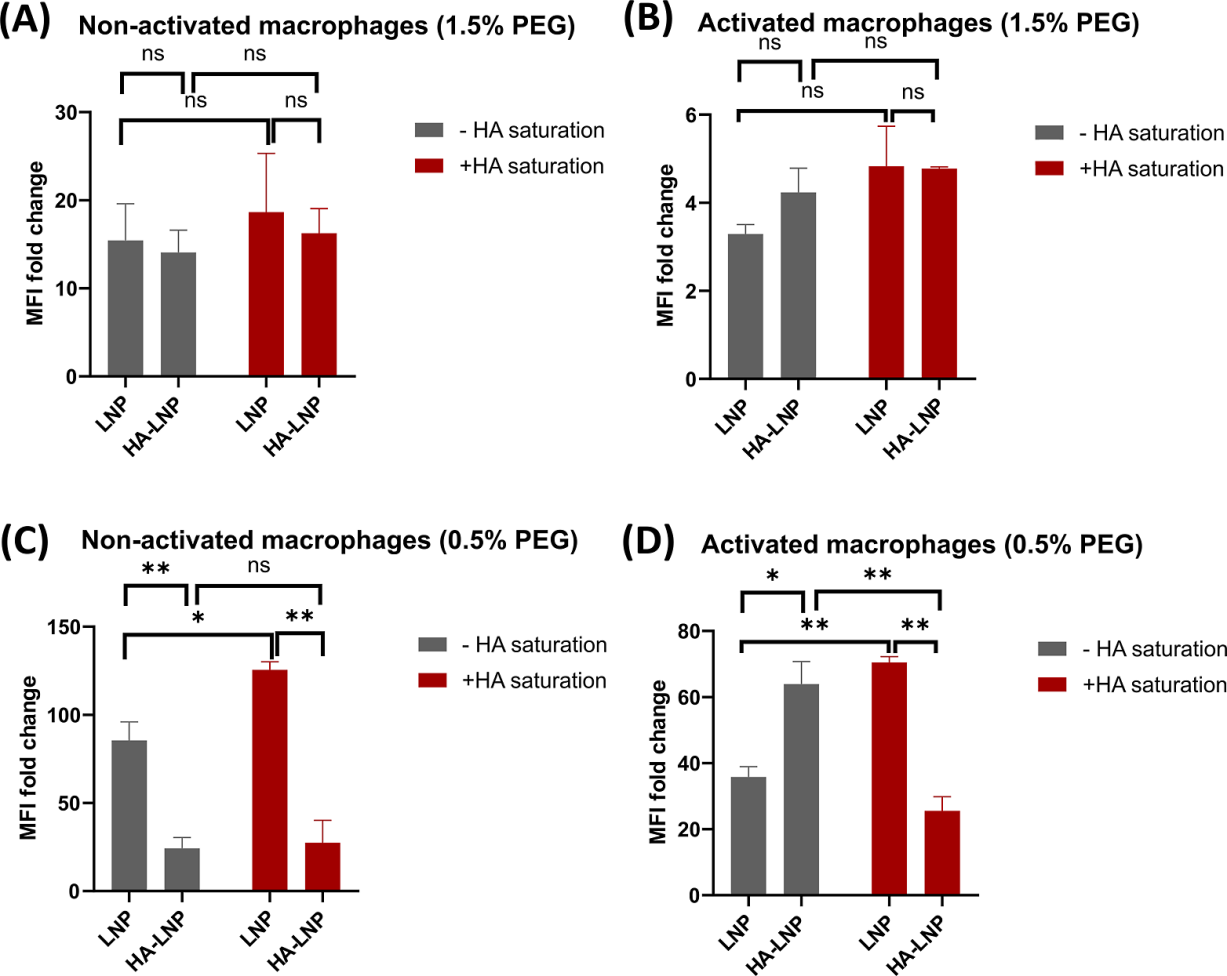
In vitro lipid nanoparticles’ uptake by flow cytometry. Cellular uptake of 1.5% PEG formulations by nonactivated macrophages (A) and activated macrophages (B) after incubation for 4 h. Cellular uptake of 0.5% PEG formulations by nonactivated macrophages (C) and activated macrophages (D) after incubation for 4 h. CD44 receptors were preblocked (red bars) for 1 h or not blocked (gray bars) by 0.5 mg/mL of free HA. * = *p* < 0.05, ** = *p* < 0.01 using two-way ANOVA. *N* = 2.

The selectivity of HA-LNP_0.5%PEG_ to CD44-overexpressing macrophages was further investigated by using confocal microscopy to assess cellular internalization and distribution. Compared to LNP_0.5%PEG_, HA-LNP_0.5%PEG_ revealed weaker rhodamine fluorescence intensities in nonactivated macrophages, whereas comparable signals in LPS-activated macrophages with upregulated CD44 expression (Figure 6), further demonstrating the selective targeting ability of HA-LNP_0.5%PEG_ to activated macrophages. Moreover, the cellular distributions of LNP_0.5%PEG_ and HA-LNP_0.5%PEG_ in activated macrophages differed. While both formulations exhibited a diffuse Cy5 fluorescence signal in the cytoplasm, indicating endosomal escape, the diffusion of LNP_0.5%PEG_ in the cytoplasmic regions was more pronounced. In contrast, HA-LNP_0.5%PEG_ exhibited a more peripheral cellular distribution on the activated macrophage edges. This observation further suggested that HA-LNP_0.5%PEG_ were taken up via CD44 receptors on the surface of the activated macrophages, which is consistent with the selectivity of HA-LNP_0.5%PEG_ toward CD44-overexpressing macrophages.

**Figure 6:**
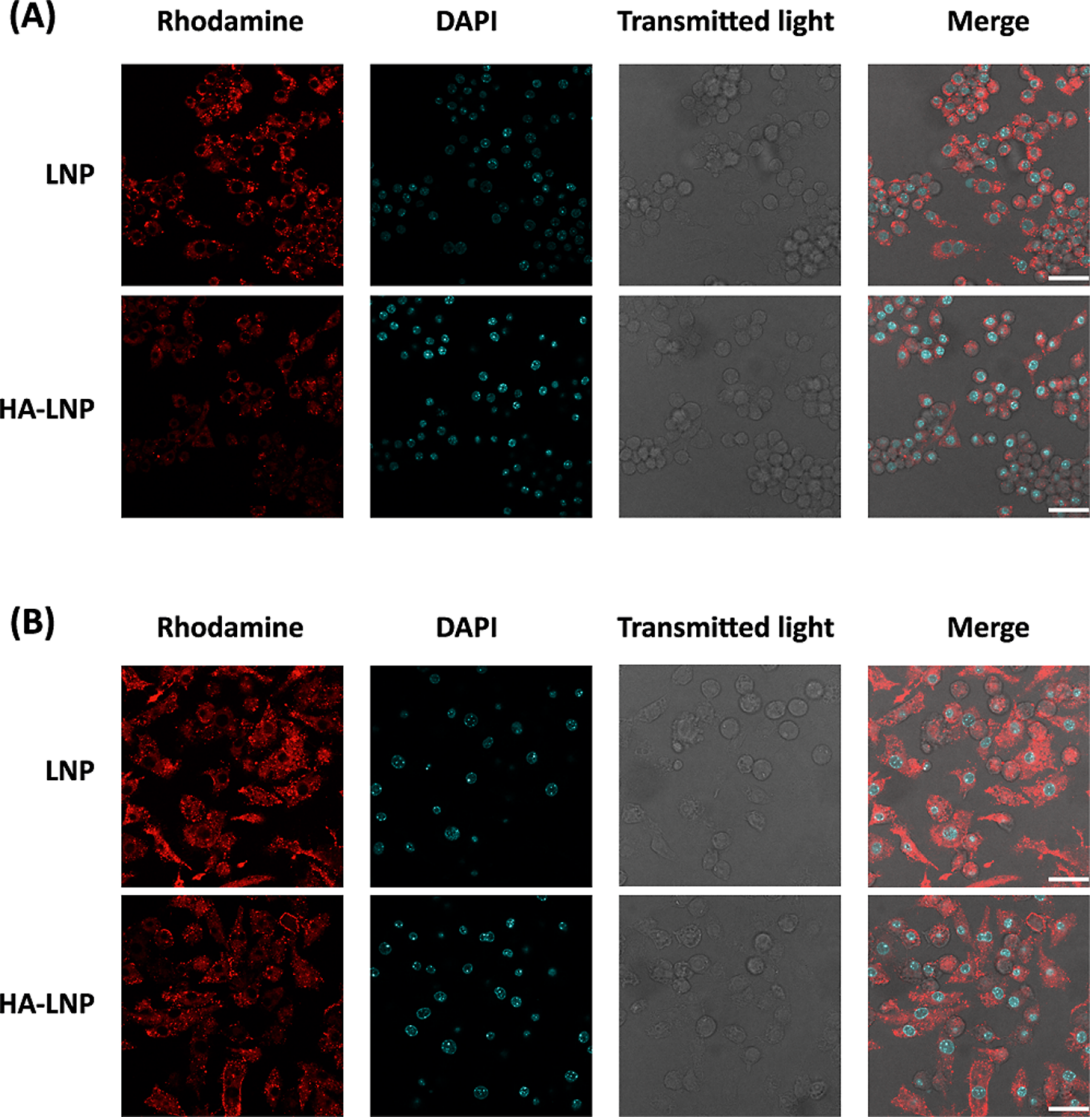
Confocal microscopy acquisitions of fixed RAW264.1 nonactivated macrophages (A) and LPS-activated macrophages (B) after incubation with LNP_0.5%PEG_ or HA-LNP_0.5%PEG_ for 4 h. LNPs were visualized with the fluorescence of rhodamine-conjugated lipids (red). Nuclei were visualized with DAPI (cyan) staining. Representative confocal images from five independent replicates are shown. Scale bar: 30 μm.

#### HA Coating Maintained the mRNA Transfection Ability

We next evaluated the LNP_0.5%PEG_ formulations using a luciferase mRNA transfection assay to determine whether the HA coating influences the mRNA transfection efficiency. Fluc mRNA was encapsulated in LNP formulations to be delivered into macrophages, where it was expected to be translated into firefly luciferase protein. After cell lysis, luciferase was released, and the substrate D-luciferin was catalyzed to produce luminescence. The resulting signal, which reflects the mRNA transfection efficiency, was measured and normalized to the total protein amount in cells. When macrophages were incubated with either LNP_0.5%PEG_ or HA-LNP_0.5%PEG_, both formulations exhibited dose-dependent luciferase expression, with higher mRNA doses resulting in increased signals of Fluc activity (Figure 7). Compared to uncoated LNPs, HA-LNP_0.5%PEG_-treated cells exhibited reduced mRNA transfection efficiency in nonactivated macrophages, while a comparable Fluc signal was observed in activated macrophages, showing that HA coating selectively delivered mRNA into CD44-overexpressed cells while maintaining the mRNA transfection efficiency. Additionally, no cytotoxicity was observed at the tested concentrations (≤0.5 μg/mL of total lipids) in either activated or nonactivated macrophages (Figure S4).

**Figure 7:**
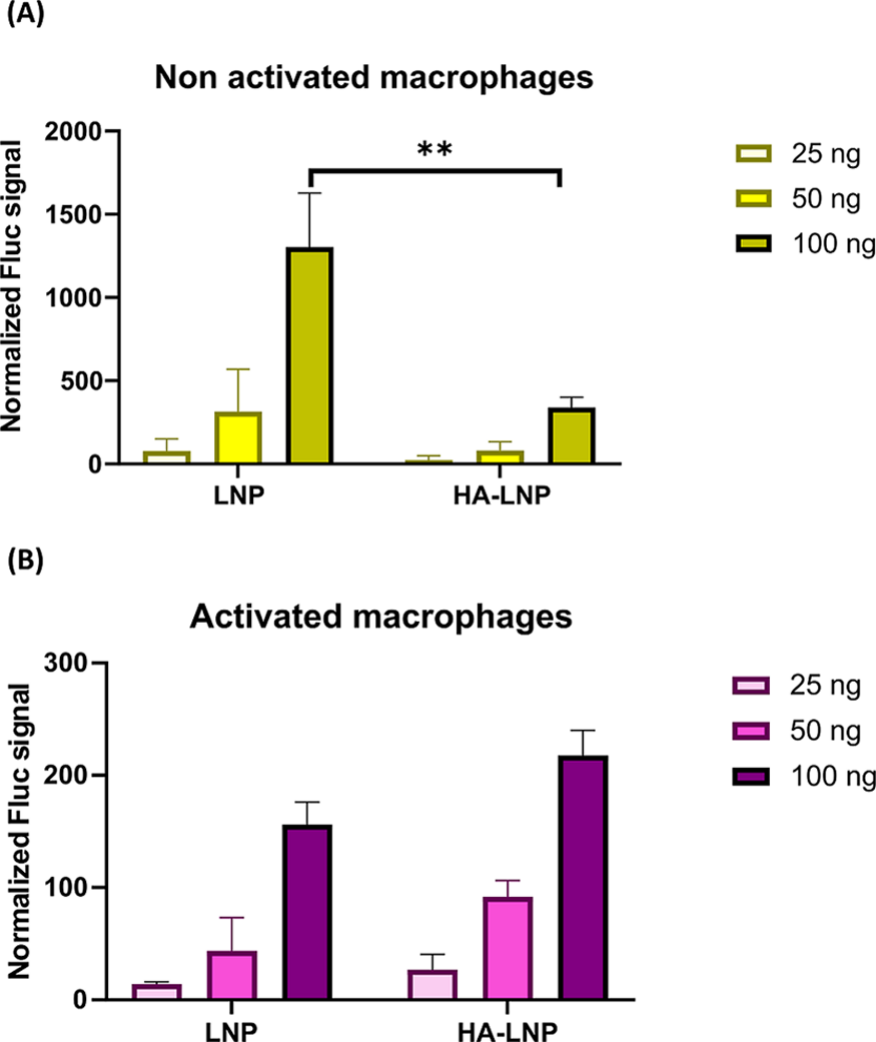
Fluc mRNA transfection efficiency by LNP_0.5% PEG_ or HA-LNP_0.5% PEG_ in nonactivated macrophages (A) and in LPS-activated macrophages (B) after incubation for 24 h. ** = *p* < 0.01 using two-way ANOVA. *N* = 2.

The influence of targeting ligand functionalization on cellular binding, internalization, mRNA endosomal release, and translation is still controversial. In most cases, targeting ligands confer nanoparticles with better selectivity and improved cellular binding/uptake. However, such an enhanced cellular uptake is not always accompanied by enhanced mRNA transfection. Our data demonstrate that HA-LNP and LNP display strong transfection efficiency, indicating an efficient cytoplasmic release of mRNA. However, despite a better uptake of HA-LNP by activated macrophages, the transfection efficiency of both formulations was similar. One possible explanation could be that CD44-mediated internalization induces a slower intracellular trafficking route than nonspecific uptake pathways.^44–46^ Since activated macrophages exhibited peripheral localization of HA-LNP compared to the LNP that had already diffused into the cytoplasm at the same time point (Figure 6B), this delayed internalization could contribute to unimproved transfection. In addition, the internalization of HA-coated nanoparticles mediated by CD44 is also associated with complex processes that involve receptor tracking on the cell surface and CD44 clustering,^47,48^ which may contribute to the slower internalization and thus delay mRNA transfection. The second explanation is that the ligand coating may impede the release of mRNA from the endosome. Efficient mRNA delivery requires an endosomal escape, which is facilitated by the deprotonation of ionizable lipids and their subsequent disruption of the endosomal membrane.^49^ It has been reported that the coating density of targeting ligands impacts the mRNA transfection efficiency.^9,50^ HA (300–500 kDa) that we used here, like PEG, is a long-chain polymer. The coating on the LNP surface may hinder the interactions between LNPs and endosomal membranes, thereby limiting the release of mRNA into the cytoplasm. This may result in the mRNA being retained within the LNP or directed to lysosomes for degradation, reducing the transfection efficiency.

Nevertheless, in our studies, the HA-coated LNPs with a reduced PEG content maintained an mRNA transfection efficiency in activated macrophages that was comparable to that of uncoated LNPs. On one side, HA confers LNPs with the ability to target activated macrophages selectively, but it requires a reduced PEG content to be effective. On the other hand, the reduced PEG content in the formulations also weakens the nanoparticles’ colloidal stability, which may facilitate the mRNA’s release from the endosome. These results highlighted the importance of balancing colloidal stability, targeting ability, and transfection efficiency by modulating the surface functionalization and PEG content to optimize LNP formulations for mRNA delivery to active macrophages.

## Conclusions

In this study, we have designed and prepared HA-coated LNPs to deliver mRNA selectively to CD44-overexpressing activated macrophages. We have synthesized HA–DPPE and chosen the postinsertion method to functionalize LNPs, which exhibited high HA-coating efficiency. By optimizing the PEG content in the LNP formulations (from 1.5% to 0.5%, n/n), we found that HA-LNP_0.5%PEG_ exhibited selectivity for CD44-overexpressed activated macrophages, with enhanced cellular uptake mediated by CD44 receptors. HA-LNP_0.5%PEG_ maintained the mRNA transfection efficiency in activated macrophages. Our findings suggest that HA functionalization, combined with a reduced PEG proportion, can deliver mRNA selectively to CD44-overexpressing activated macrophages via LNPs. This study offers a novel perspective on the targeted treatment of inflammatory macrophages.

## Supplementary Material



## Notes

Purification and identification of HA-LNPs; cryo-TEM imaging of LNPs and HA-LNPs; gating strategy for flow cytometry; and MTT-based in vitro cell viability assay (PDF)

## Abbreviations

proton nuclear magnetic resonance: (^1^H NMR)
1,2-dioleoyl-*sn*-glycero-3-phosphoethanolamine-*N*-(lissamine rhodamine B sulfonyl) (ammonium salt): (18:1 Liss Rhod-PE);
C–C motif chemokine ligand 2: (CCL-2)
cluster of differentiation 44: (CD44)
cryo-transmission electron microscopy: (Cryo-TEM)
C-X-C motif) ligand 8: (CXCL-8)
cyanine 5: (Cy5)
deuterium oxide: (D_2_O)
damage-associated molecular patterns: (DAMPs)
4,6-diamidino-2-phenylindole: (DAPI)
dynamic light scattering: (DLS)
1,2-dimyristoyl-rac-glycero-3-methoxypolyethylene glycol-2000: (DMG-PEG_2000_)
dimethyl sulfoxide: (DMSO)
1,2-di­(9Z-octadecenoyl)-*sn*-glycero-3-phosphocholine: (DOPC)
1,2-dihexadecanoyl-*sn*-glycero-3-phosphoethanolamine: (DPPE)
1-ethyl-3-(3-(dimethylamino)­propyl) carbodiimide hydrochloride: (EDC)
encapsulation fraction: (EF)
encapsulation yield: (EY)
Food and Drug Administration: (FDA)
forward scatter: (FSC)
firefly luciferase: (Fluc)
hyaluronic acid: (HA)
HA-coated LNPs: (HA-LNPs)
interleukin-6: (IL-6)
dissociation constant: (*K*_D_)
lipid nanoparticles: (LNPs)
lipopolysaccharide: (LPS)
mean fluorescence intensity: (MFI)
messenger RNA: (mRNA)
thiazolyl blue tetrazolium bromide: (MTT)
molecular weight: (*M*_W_)
*N*-hydroxysuccinimide: (NHS)
pathogen-associated molecular patterns: (PAMPs)
phosphate-buffered saline: (PBS)
polydispersity index: (PDI)
polyethylene glycol: (PEG)
paraformaldehyde: (PFA)
photomultiplier tube: (PMT)
DPPE substitution degree: (SD_DPPE_)
side scatter: (SSC)
*Tris*-borate-ethylenediaminetetraacetic acid: (TBE)
tumor necrosis factor α: (TNF-α)
mean hydrodynamic diameter: (*Z*-average)
