# Supplementary material for: Selective mRNA Delivery to Activated Macrophages via Hyaluronic Acid-Functionalized Lipid Nanoparticles with Optimized PEGylation
Source: Biomacromolecules. 2026 Feb 10;27(3):2078–90. doi: 10.1021/acs.biomac.5c02390 (PMC12977047; doi:10.1021/acs.biomac.5c02390)
Supplement: Supplementary file 1 [file bm5c02390_si_001.pdf]

Selective mRNA Delivery to Activated Macrophages via Hyaluronic Acid-Functionalized Lipid Nanoparticles with Optimized PEGylation

Supplementary information

Mengyuan Cao, [†] François Fay, [†,‡] Adrouchan Hotier,[†] Séverine Domenichini,[§] Lucile Alexandre, [//] Christopher Ribes, [//] Florence Gazeau, [//] Hervé Hillaireau,[*,†] and Elias Fattal,[*,†]

[†] Institut Galien Paris−Saclay, UMR CNRS 8612, Université Paris−Saclay, 91400 Orsay, France

[‡] Institut Universitaire de France (IUF), 75005 Paris, France

[§] Plateforme MIPSIT-Ingénierie et Plateformes au Service de l'Innovation Thérapeutique, UMS-IPSIT Université Paris-Saclay, US 31 INSERM, UAR 3679 CNRS , 91400 Orsay, France

[//] Laboratoire NABI NAnomédecine, Biologie extracellulaire, Intégratome et Innovations, Université Paris Cité, CNRS UMR8175, INSERM U1334, 75006 Paris, France

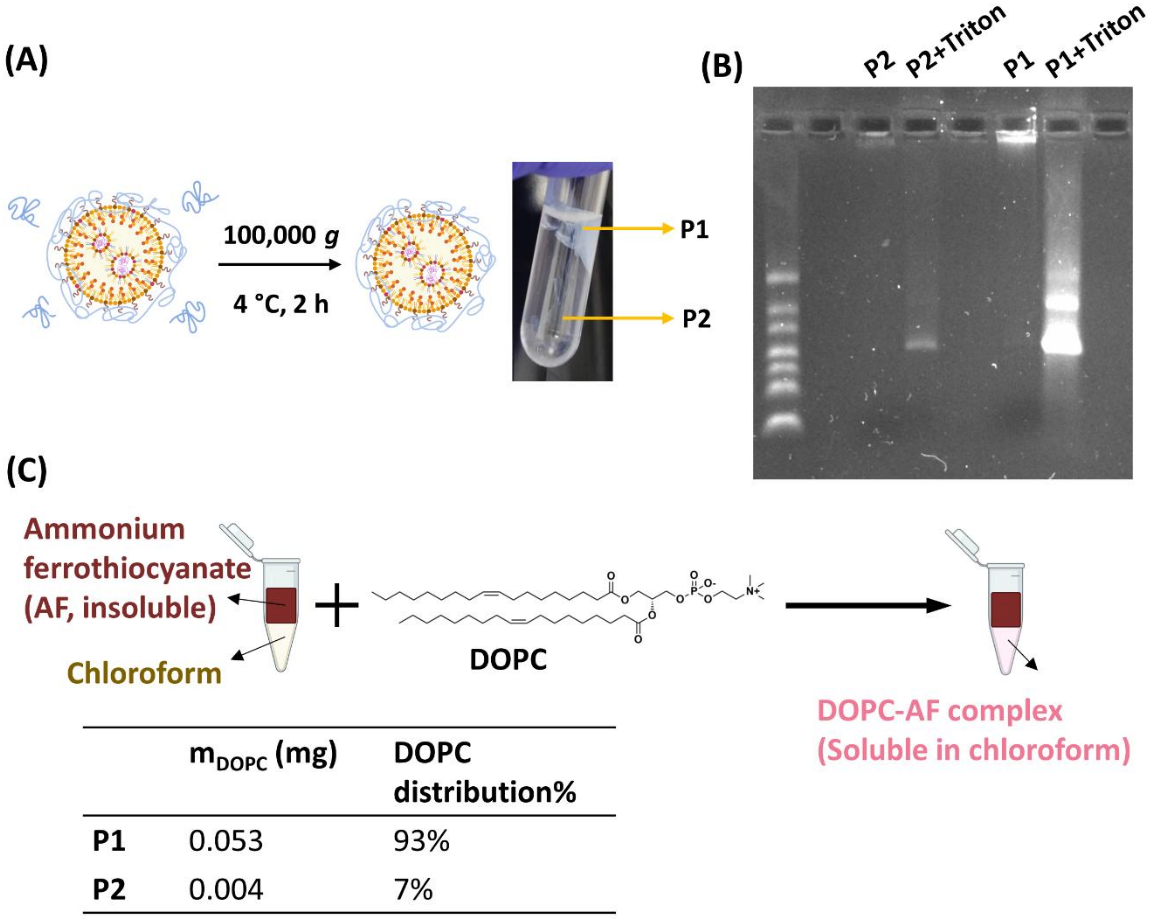

	m_{DOPC} (mg)	DOPC distribution%
**P1**	0.053	93%
**P2**	0.004	7%

Figure S 1. Purification and identification of HA-LNPs. (A) schematic representation of ultracentrifugation conditions to purify free HA-DPPE from HA-LNPs and subsequent phase separation into an upper, opaque phase (P1) and a lower, transparent phase (P2). (B) Representative agarose gel electrophoresis of samples from P1 and P2 treated or no with Triton X-100. (C) Phospholipid quantification of samples from two phases by Stewart's method.

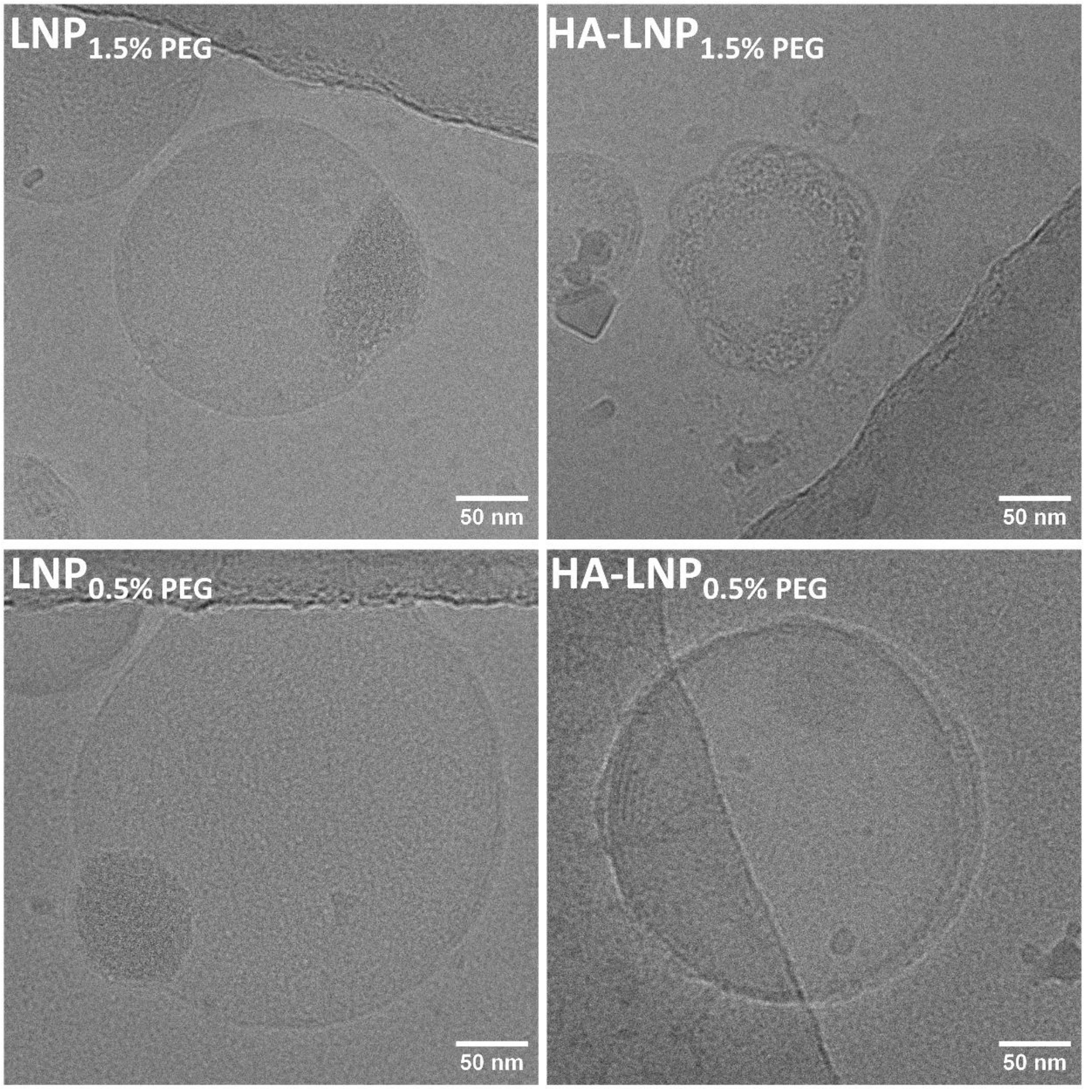

Figure S 2. Cryo-TEM imaging of LNPs and HA-LNPs, containing 0.5% or 1.5% PEG. Scale bars = 50 nm.

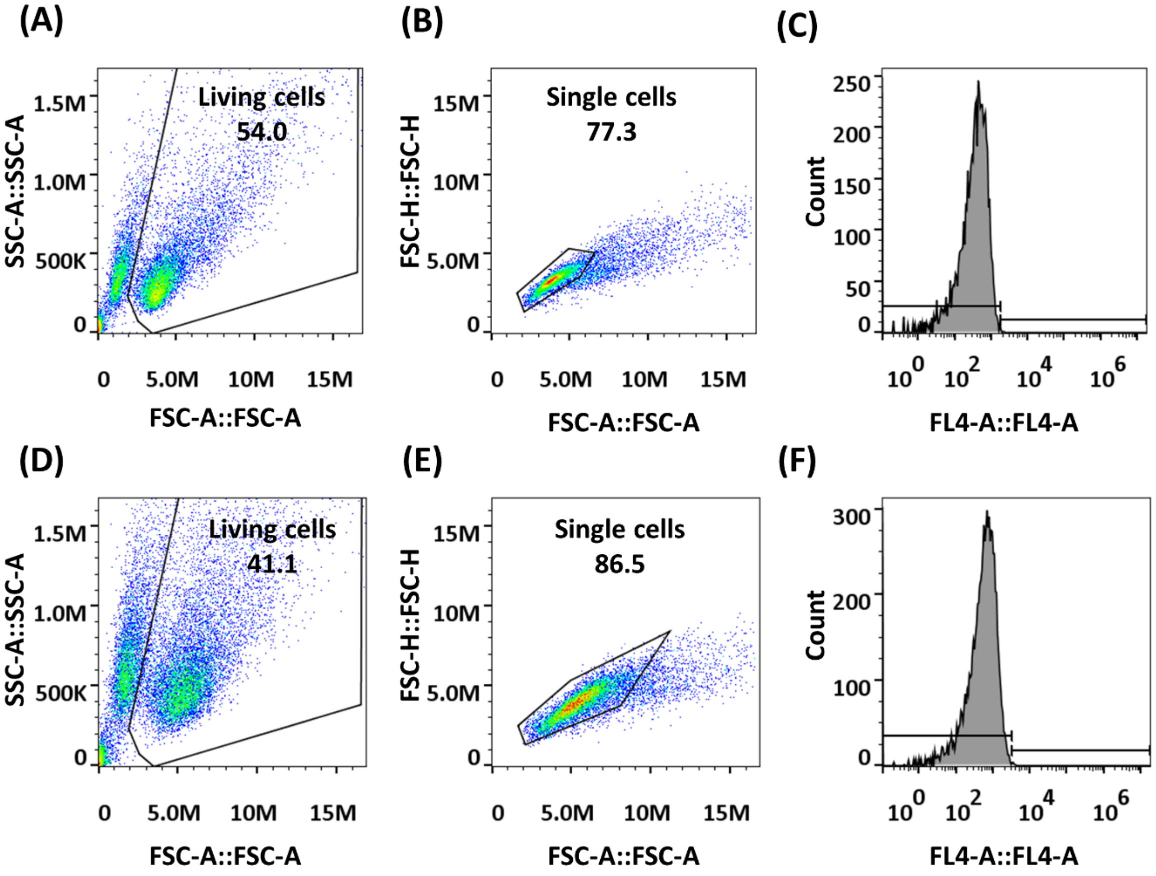

Figure S3. Gating strategy to define anti-CD44 antibody negative and positive populations in non-activated macrophages (A, B and C) and in LPS-activated macrophages (D, E and F). (A, D) Living cells; (B, E) single cells; (C, F) Rhod-PE negative cells.

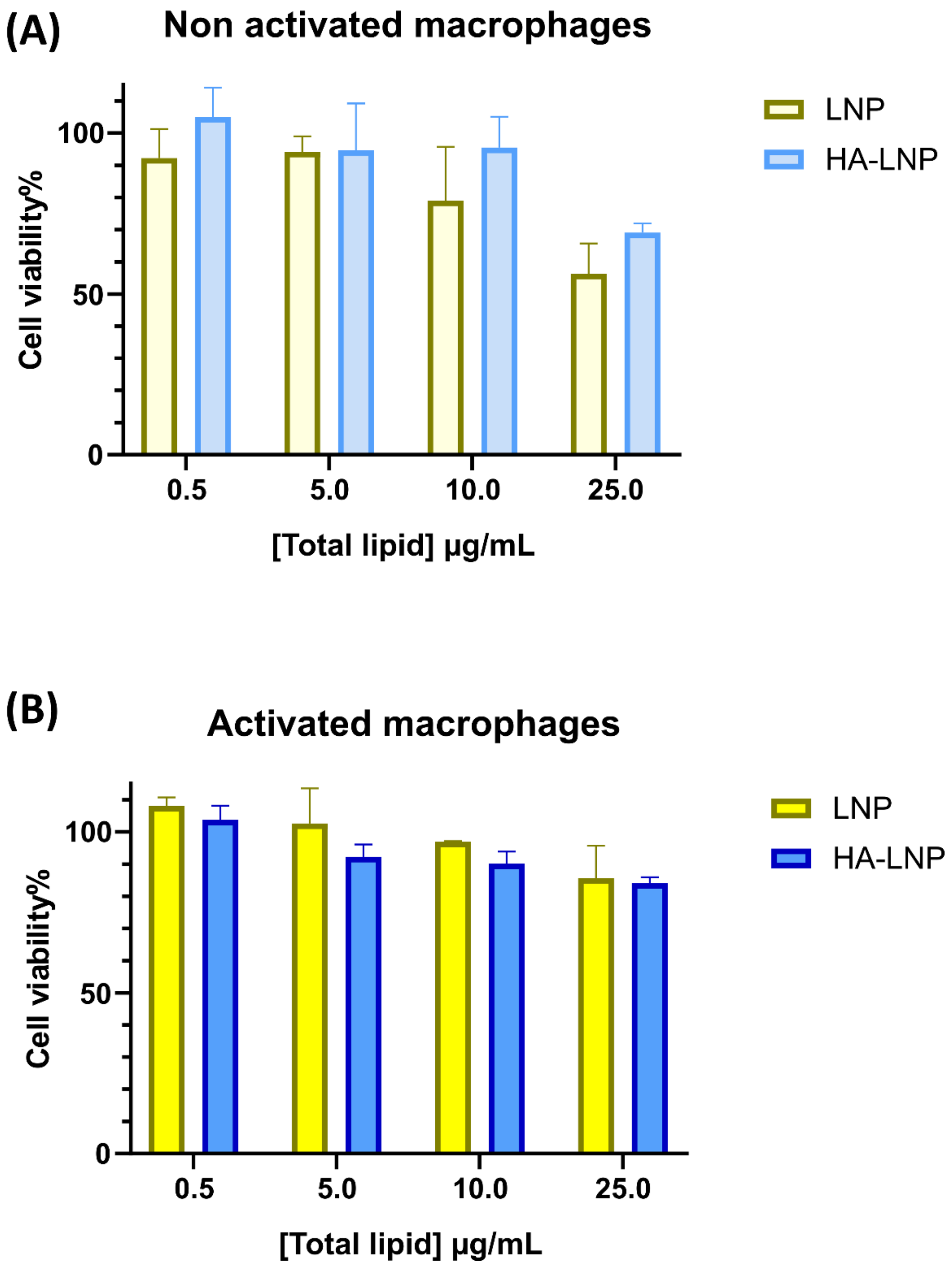

Figure S4. MTT-based *in vitro* cell viability assay on non-activated macrophages (A) or LPS-activated macrophages (B) treated with $LNP_{0.5\%PEG}$ or $HA\text{-}LNP_{0.5\%PEG}$ (N = 3).
